# Young Adult Case of Fontan-associated Liver Disease with Hepatocellular Carcinoma During the Transition from Pediatric to Internal Medicine Care and Follow-up

**DOI:** 10.14789/jmj.JMJ22-0037-CR

**Published:** 2023-04-26

**Authors:** HIDEO FUKUNAGA, MITSUYOSHI SUZUKI, KEIYA SATO, SAKIKO MIYAZAKI, AKIRA UCHIYAMA, SHUNPEI YAMASHINA, MAMIKO MIYASHITA, KEN TAKAHASHI, TOSHIAKI SHIMIZU

**Affiliations:** 1 Department of Pediatrics, Juntendo University Faculty of Medicine, Tokyo, Japan; 1 Department of Pediatrics, Juntendo University Faculty of Medicine, Tokyo, Japan; 2 Department of Cardiology, Juntendo University Faculty of Medicine, Tokyo, Japan; 2 Department of Cardiology, Juntendo University Faculty of Medicine, Tokyo, Japan; 3 Department of Gastroenterology, Juntendo University Faculty of Medicine, Tokyo, Japan; 3 Department of Gastroenterology, Juntendo University Faculty of Medicine, Tokyo, Japan; 4 Department of Hepatobiliary-Pancreatic Surgery, Juntendo University Faculty of Medicine, Tokyo, Japan; 4 Department of Hepatobiliary-Pancreatic Surgery, Juntendo University Faculty of Medicine, Tokyo, Japan

**Keywords:** Fontan-associated liver disease, Fontan circulation, liver fibrosis, hepatocellular carcinoma, transitional care

## Abstract

In recent years, the outcomes of the Fontan procedure have been good, but Fontan-associated liver disease (FALD), which causes congestive hepatopathy due to elevated central venous pressure (CVP), has become a serious problem when considering patients' long-term prognosis. A 28-year-old woman with Emanuel syndrome was admitted to our hospital for the treatment of hepatocellular carcinoma (HCC). She was diagnosed with pulmonary atresia and underwent a bidirectional pulmonary artery shunt at the age of 1 year and 10 months and the Fontan procedure at 4 years of age. Blood tests showed an increase in γ-glutamyltransferase in her early 20s and a marked increase in alfa-fetoprotein levels at age 27 years. She was diagnosed as having HCC in the S7 region by contrast-enhanced computed tomography and underwent hepatectomy. There were no serious adverse events, and the patient has survived 18 months after surgery without recurrence. In this report, the optimal time for the transition from the pediatrics department to adult healthcare units is also discussed, along with the management system for FALD in our hospital.

## Introduction

Though advances in pediatric care have saved many lives, an increasing number of patients are coming of age with coexisting chronic diseases. At the later stages of Fontan surgery for complex congenital heart malformations, it has become apparent that patients present with cardiovascular, as well as hepatic-gastrointestinal, complications^1)^. Currently, Fontan-associated liver disease (FALD) caused by congestive hepatopathy, which is characterized by high central venous pressure (CVP) in the Fontan circulation compared with the normal heart, is one of the most severe problems^2, 3)^. Not only liver fibrosis, but also cirrhosis, localized nodular hyperplasia, hepatic adenoma, and hepatocellular carcinoma (HCC), which are defined as FALD, have been reported in the remote period after the Fontan operation^4, 5)^. In 1990, a case of HCC associated with cardiac cirrhosis was first recognized by Ho et al.^6)^. Fifteen years later, Ghaferi et al. reported the second case with HCC after the Fontan procedure^7)^. In the last 7-8 years, the incidence of HCC after Fontan surgery has become widely known, and the number of publications on this topic has been increasing.

At present, approximately 400 Fontan procedures are performed annually in Japan, and more than 1,000 cases are performed annually in the United States. Over the past several decades, various improvements in surgical techniques and perioperative management have significantly reduced perioperative and early postoperative mortality^8)^, and the average life expectancy of patients with underlying serious congenital heart disease has improved from 17 years in 2000 to 25 years in 2010^9, 10)^. Consequently, liver management for FALD surveillance is important based on this background, and transitional care from pediatrics to internal medicine is gradually recognized these days. In order to build smooth transitional care for FALD surveillance, it is necessary to establish a coordinated care system for cardiovascular and liver diseases from the early postoperative period. A case of FALD in a young adult with HCC that was successfully resected due to early detection of the tumor is reported, along with a discussion of the management system for FALD in our hospital.

## Case presentation

A 28-year-old woman who had been born at a gestational age of 41 weeks and 5 days weighing 2,416 grams had cyanosis and a heart murmur, and the patient was transferred to our hospital on day 5. Echocardiography showed pulmonary atresia with an intact ventricular septum, total anomalous pulmonary venous return type IIa, atrial septal defect, patent ductus arteriosus, absent right superior vena cava, and persistent left superior vena cava. Based on these clinical symptoms, chromosomal analysis was performed and showed Emanuel syndrome [47XX, dic (22) (q11.2)]. Her history of heart surgery was as follows: balloon dilation of the ductus arteriosus at 2 months of age; left-sided systemic to pulmonary shunt (Blalock-Taussig shunt) and modified Brock's operation at 4 months of age; bidirectional cavo-pulmonary shunt, division of the PDA, and division of the left-sided Blalock-Taussig shunt at 1 year and 10 months of age; and the Fontan procedure using a 16-mm Gore-Tex extracardiac conduit at age 4 years and 2 months.

**Figure 1:**
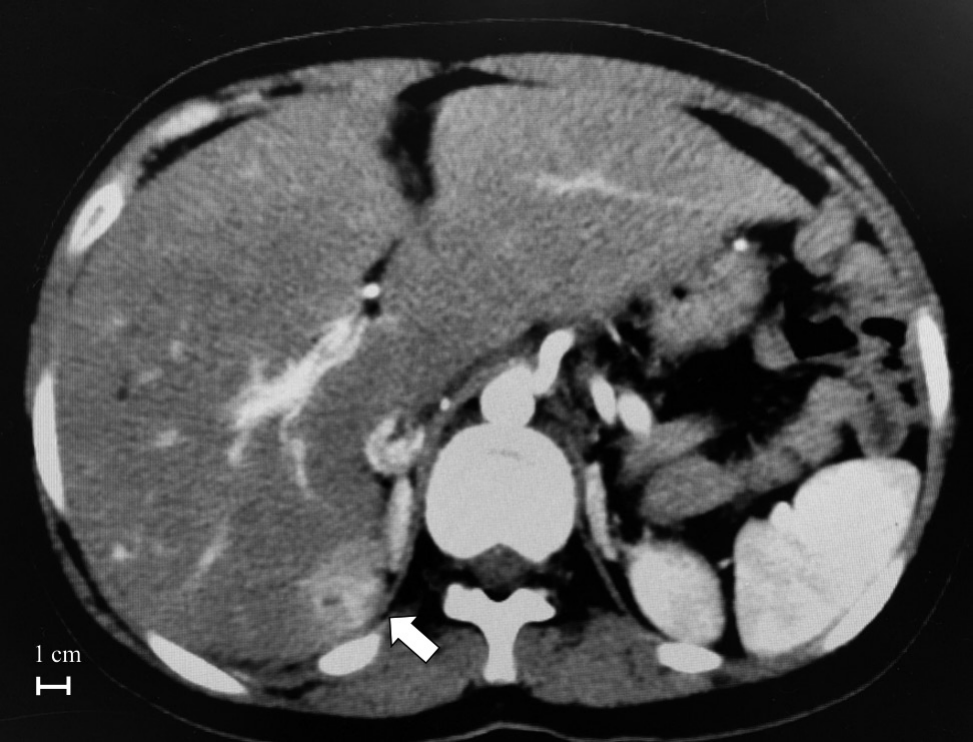
Contrast-enhanced CT of the abdomen. A mass with contrast effect is seen in the S7 area of the right lobe of the liver (arrow), which was suspected to be hepatocellular carcinoma. The maximum diameter of the tumor is 27 mm.

Since the patient remained asymptomatic for more than 20 years thereafter, her follow-up was conducted only by pediatric cardiologists. When she was over 20 years of age, at the time that pediatric cardiologists have widely recognized the development of HCC after the Fontan procedure, the course and prognosis of cases developing FALD and HCC were carefully explained to her family, and it was recommended that she visit the Department of Gastroenterology for adults. Her family was initially hesitant to have her seen at the Departments of Cardiology and Gastroenterology for adults because of her immature personality due to the chromosomal anomaly. Finally, her family decided that she would visit the Departments of Cardiology and Gastroenterology for adults when she was 27 years old. As soon as she was transferred to the Gastroenterology Department, progression of FALD was suspected on echosonography, and then contrast-enhanced computed tomography (CT) performed as the first screening showed a contrast-enhanced mass lesion in the S7 area of the liver (Figure 1). In addition, an elevated serum alfa-fetoprotein (AFP) level was found (772 ng/mL), strongly suggesting hepatocellular carcinoma.

**Figure 2:**
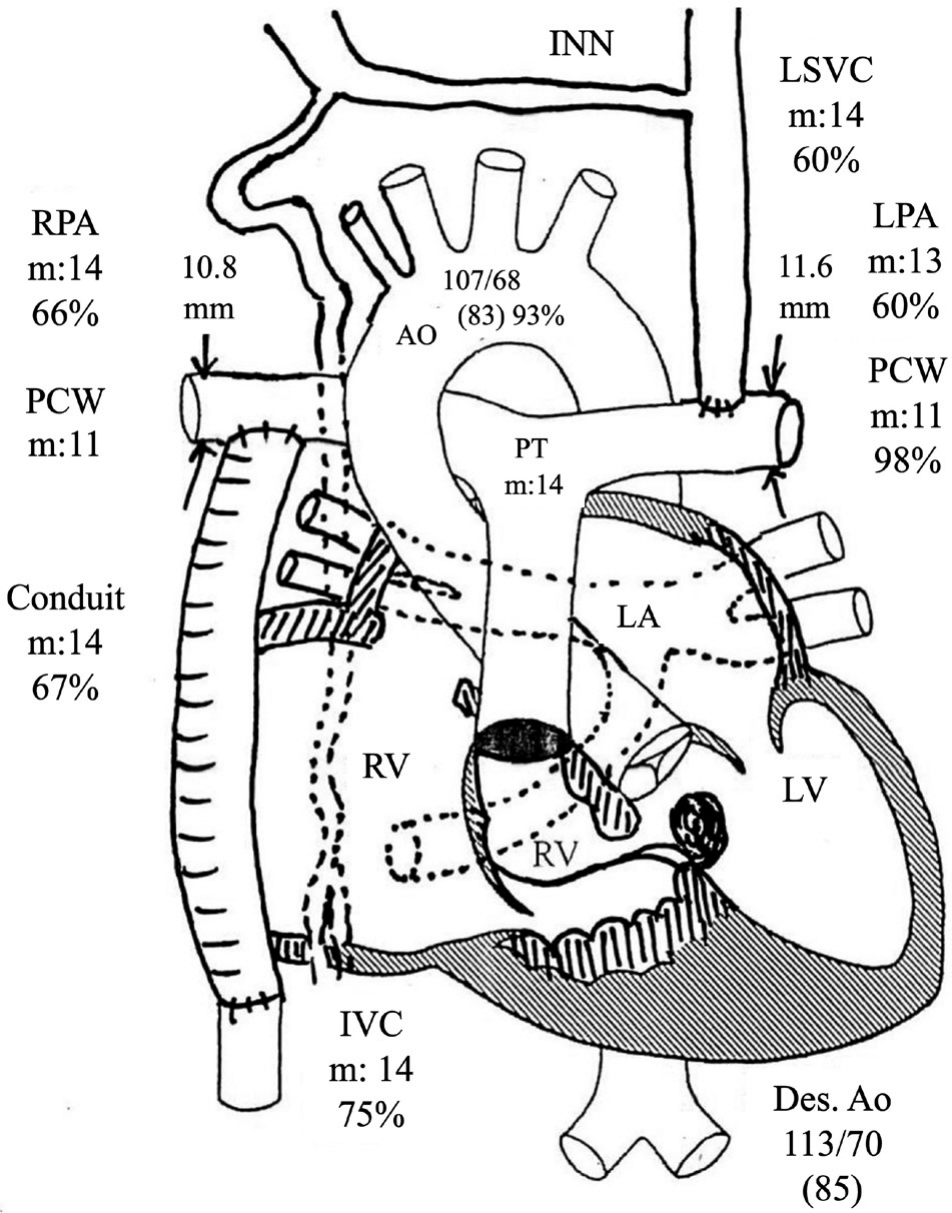
Findings of preoperative cardiac catheterization at 28 years of age Cardiac index = 3.08 L/min/m^2^, pulmonary blood flow/systemic blood flow ratio (Qp/Qs) =1.02, pulmonary vessel resistance = 0.94-1.42 Um^2^ (Wood units), pulmonary artery index = 152. INN: innominate vein, LSVC: left superior vena cava, LPA: left pulmonary artery, PCW: pulmonary capillary wedge pressure, RPA: right pulmonary artery, PT: pulmonary trunk, LA: left atrium, RV: right ventricle, LV: left ventricle, Des. Ao: descending aorta, m: mean pressure value (mmHg).

**Figure 3:**
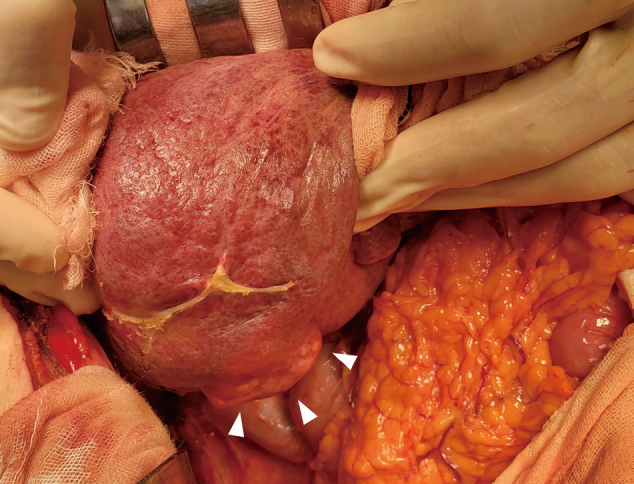
Appearance of the liver during surgery The hepatic surface is irregular and plastic, and part of the liver protrudes outside the liver. The location of the tumor is indicated by a triangle.

**Figure 4:**
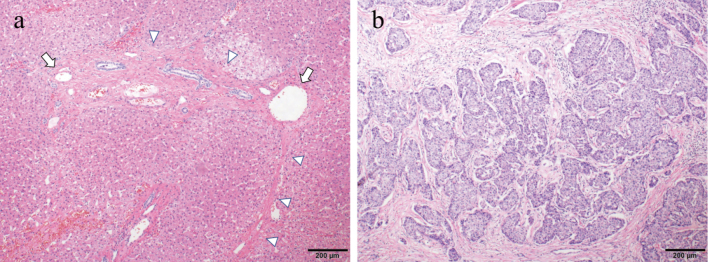
a. Pathological findings of hematoxylin and eosin staining (non-tumor site). The portal vein area is fibrotic and enlarged (arrow), and bridging fibrosis is partially observed (F3) (triangle), indicating a precirrhotic state. b. Pathological findings of hematoxylin and eosin staining (tumor site). The histopathological diagnosis of poorly differentiated hepatocellular carcinoma is confirmed.

On admission, she was in good general condition (New York Heart Association functional classification: NYHA I). The laboratory findings were as follows: hemoglobin 15.8 g/dL, platelet count 14.2 ×109/L, alanine aminotransferase 35 (13-55) U/L, *γ*-glutamyltransferase 212 (8-90) U/L, total bilirubin 1.85 (0.1-0.6) mg/dL, total protein 8.0 (5.3-7.2) g/dL, and the hepatic fibrosis marker Mac-2 binding protein glycosylation isomer (M2BPGi) 0.43 (0.00-0.99; expressed as a cutoff index). Preoperative cardiac catheterization was performed, and CVP was 14 mmHg, which was the same as that at 5 years of age (Figure 2). Surgical treatment was performed, and the tumor was completely removed. The surface of the liver was irregular and plastic, and part of the tumor protruded outside the liver (Figure 3). The histopathological findings of liver tissue obtained at operation confirmed poorly differentiated hepatocellular carcinoma (Figure 4). Her postoperative course was uneventful, with no major perioperative circulatory disturbances, resulting in her discharge on the 8th postoperative day. Eighteen months have passed since the surgery, and no re-elevation of AFP levels has been observed.

## Discussion

Until 1990, the classic Fontan procedure was performed to directly connect the right atrium to the right pulmonary artery (PA) by closing the atrial-septal defect. After 2000, an extracardiac conduit insertion connecting the inferior vena cava to the right PA was performed. With the increasing number of long-term survivors after Fontan surgery, the problem of FALD has become evident. In patients with Fontan circulation, the complication rates of liver fibrosis, liver cirrhosis, hepatocellular carcinoma, gastroesophageal varices, and protein-losing gastroenteropathy after Fontan surgery were reported to be 0.85-58.3%, 20.5-23.0%, 1.2-9.8%, 19.2-33.3%, and 3.7-24.0%, respectively^11-16)^. In addition, the estimated annual incidence of HCC is 1.5-5.0%^2)^. In a report from one of the earliest centers to perform the Fontan procedure in Japan, HCC was diagnosed at a median age of 32.5 years (range: 20.6-46.1 years), and the median time from the Fontan procedure to diagnosis was 21.3 years (3.7-31.2 years)^12)^. This is similar to a report from Western countries, in which the mean age at HCC diagnosis was 30.0 ± 9.4 years, and the mean interval was 21.6 ± 7.4 years^17)^. Generally, the long-term course of Emanuel syndrome, characterized by multiple congenital anomalies and craniofacial dysmorphism, has not been well understood, since reports about patients are mainly from infancy and early childhood^18)^. Of note, the relationship between carcinogenesis and Emanuel syndrome may also be unclear. In the present case, HCC was observed 24 years after Fontan surgery, which is a common period for the diagnosis of HCC after Fontan surgery, suggesting the postoperative period after Fontan surgery may have influenced the pathogenesis of HCC.

**Figure 5:**
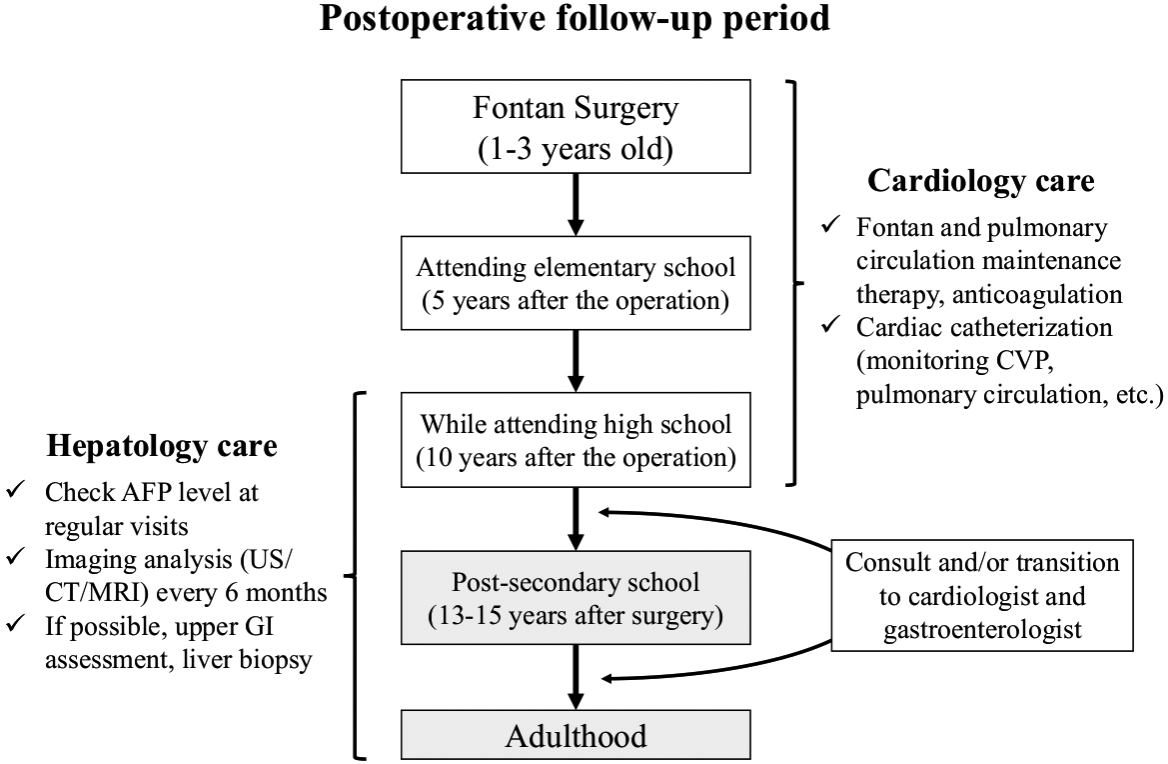
Transitional care and FALD-HCC surveillance CVP: central venous pressure, AFP: alfa-fetoprotein, US: ultrasonography, CT: computed tomography, MRI: magnetic resonance imaging, GI: gastrointestinal.

Transitional care can be classified into three categories: (1) complete transition from pediatrics to internal medicine; (2) pediatrics continues to treat congenital diseases and disorders, while internal medicine takes over for health problems specific to adulthood; and (3) pediatrics alone continues to treat patients when no appropriate adult care department is available^19)^. Currently, adult departments are usually specialized, and multiple referrals should be made in cases of multisystem syndromes or complications. In the present case, transitional care was practiced according to pattern (2). Regardless of the optimal transition time from the age of 10 to the early 20s, bridging to specialists in adult departments at an appropriate time according to the natural course of the disease and family needs would be considered a possible form of transitional care. A nationwide survey was conducted to determine the epidemiology of FALD from 2021 to 2022 by a research group of the Japanese Ministry of Health, Labour and Welfare, which will provide the basic data for the creation of a transitional care for FALD surveillance protocol in the future (https://mhlw-grants.niph.go.jp/project/ 147343, accessed on 27 Jan 2023). Since no official guidelines have yet been provided, the management system for postoperative Fontan patients at our institution has been developed (Figure 5). Persistently elevated AFP is well known to be a risk biomarker for the development of HCC in patients without a background of the Fontan procedure^20)^. In patients who underwent the Fontan procedure, AFP levels can be measured together with routine blood tests and are likely to be useful as screening for HCC. However, approximately one-quarter of post-Fontan patients diagnosed with HCC showed normal AFP values, retrospectively, suggesting the importance of imaging analysis^17)^. Especially in patients with mental retardation, as in the present case, the CT scan may require sedation and thus may not be easily performed. However, based on previous reports^2, 12, 17)^, CT or magnetic resonance imaging should be recommended to screen for HCC in patients more than 10 years after the procedure.

Pediatric cardiologists and thoracic surgeons need to be aware that FALD is the precursor of HCC in the Fontan circulation as general knowledge and should inform patients and their families of these risks before the operation. In addition, to monitor carcinogenesis, regular check-ups of tumor markers such as AFP combined with imaging analysis would be helpful even in childhood. Further studies should focus on creating a protocol for liver surveillance after Fontan surgery.

## Conclusion

A young adult patient with FALD-related HCC who was successfully treated by surgical treatment was described. After the Fontan procedure, it is necessary to provide continuous postoperative care considering complications such as FALD. Thus, it is crucial to build a transitional care system that smoothly bridges the gap from childhood to adulthood.

## Informed consent to participate

The patient's guardians provided written informed consent for publication of the case details and analysis.

## Funding

The authors received no external funding for this study.

## Author contributions

HF and MS wrote the manuscript and created the figure. KS, SM, AU, and MM treated the patient during hospitalization and conducted follow-up at the outpatient clinic. KT and TS supervised the study and revised the manuscript. All authors approved the manuscript prior to submission.

## Conflicts of interest statement

The authors have no conflicts of interest to disclose.

## References

[1] Warnes CA, Williams RG, Bashore TM, et al: ACC/AHA 2008 guidelines for the management of adults with congenital heart disease: a report of the American College of Cardiology/American Heart Association Task Force on Practice Guidelines (Writing Committee to Develop Guidelines on the Management of Adults With Congenital Heart Disease). Developed in Collaboration With the American Society of Echocardiography, Heart Rhythm Society, International Society for Adult Congenital Heart Disease, Society for Cardiovascular Angiography and Interventions, and Society of Thoracic Surgeons. J Am Coll Cardiol. 2008; 52: e143-e263. 10.1016/j.jacc.2008.10.001 19038677

[2] Kogiso T, Tokushige K: Fontan-associated liver disease and hepatocellular carcinoma in adults. Sci Rep. 2020; 10: 21742. 10.1038/s41598-020-78840-y PMC7728791 33303924

[3] Gordon-Walker TT, Bove K, Veldtman G: Fontan-associated liver disease: A review. J Cardiol. 2019; 74: 223-232. 10.1016/j.jjcc.2019.02.016 30928109

[4] Kogiso T, Sagawa T, Taniai M, et al: Risk factors for Fontan-associated hepatocellular carcinoma. PLoS One. 2022; 17: e0270230. 10.1371/journal.pone.0270230 PMC9205474 35714161

[5] Kuwabara M, Niwa K, Toyoda T, et al: Liver Cirrhosis and/or Hepatocellular Carcinoma Occurring Late After the Fontan Procedure- A Nationwide Survey in Japan. Circ J. 2018; 82: 1155-1160. 10.1253/circj.CJ-17-1053 29445059

[6] Ho SS, Brown R, Fitzgibbon B: Hepatocellular carcinoma with cardiac cirrhosis. Med J Aust. 1990; 152: 553-554. 10.5694/j.1326-5377.1990.tb125362.x 2160048

[7] Ghaferi AA, Hutchins GM: Progression of liver pathology in patients undergoing the Fontan procedure: Chronic passive congestion, cardiac cirrhosis, hepatic adenoma, and hepatocellular carcinoma. J Thorac Cardiovasc Surg. 2005; 129 : 1348-1352. 10.1016/j.jtcvs.2004.10.005 15942576

[8] de Leval MR, Deanfield JE: Four decades of Fontan palliation. Nat Rev Cardiol. 2010; 7: 520-527. 10.1038/nrcardio.2010.99 20585329

[9] Marelli AJ, Mackie AS, Ionescu-Ittu R, Rahme E, Pilote L: Congenital heart disease in the general population: changing prevalence and age distribution. Circulation. 2007; 115: 163-172. 10.1161/CIRCULATIONAHA.106.627224 17210844

[10] Daniels CJ, Bradley EA, Landzberg MJ, *et al*: Fontan-Associated Liver Disease: Proceedings from the American College of Cardiology Stakeholders Meeting, October 1 to 2, 2015, Washington DC. J Am Coll Cardiol. 2017; 70: 3173-3194. 10.1016/j.jacc.2017.10.045 29268929

[11] Goldberg DJ, Surrey LF, Glatz AC, et al: Hepatic Fibrosis Is Universal Following Fontan Operation, and Severity is Associated With Time From Surgery: A Liver Biopsy and Hemodynamic Study. J Am Heart Assoc. 2017; 6: e004809. 10.1161/JAHA.116.004809 PMC5524062 28446492

[12] Sagawa T, Kogiso T, Sugiyama H, Hashimoto E, Yamamoto M, Tokushige K: Characteristics of hepatocellular carcinoma arising from Fontan-associated liver disease. Hepatol Res. 2020; 50: 853-862. 10.1111/hepr.13500 32219953

[13] Schwartz MC, Sullivan LM, Glatz AC, et al: Portal and sinusoidal fibrosis are common on liver biopsy after Fontan surgery. Pediatr Cardiol. 2013; 34: 135-142. 10.1007/s00246-012-0402-9 22695765

[14] Rodriguez De Santiago E, Tellez L, Guerrero A, Albillos A: Hepatocellular carcinoma after Fontan surgery: A systematic review. Hepatol Res. 2021; 51: 116-134. 10.1111/hepr.13582 33037858

[15] Elder RW, McCabe NM, Hebson C, et al: Features of portal hypertension are associated with major adverse events in Fontan patients: the VAST study. Int J Cardiol. 2013; 168: 3764-3769. 10.1016/j.ijcard.2013.06.008 PMC3805740 23849105

[16] Kiesewetter CH, Sheron N, Vettukattill JJ, et al: Hepatic changes in the failing Fontan circulation. Heart. 2007; 93: 579-584. 10.1136/hrt.2006.094516 PMC1955554 17005713

[17] Possner M, Gordon-Walker T, Egbe AC, et al: Hepatocellular carcinoma and the Fontan circulation: Clinical presentation and outcomes. Int J Cardiol. 2021; 322: 142-148. 10.1016/j.ijcard.2020.08.057 32828959

[18] Carter MT, St Pierre SA, Zackai EH, et al: Phenotypic delineation of Emanuel syndrome (supernumerary derivative 22 syndrome): Clinical features of 63 individuals. Am J Med Genet A. 2009; 149A: 1712-1721. 10.1002/ajmg.a.32957 PMC2733334 19606488

[19] Yokoya S, Ochiai R, Kobayashi N, et al: Japan Pediatric Society Working Group on Transitional Patients. Recommendations for transitional care for patients with childhood-onset diseases. J Jpn Pediatr Soc. 2014; 118: 98-106. (in Japanese)

[20] Tsukuma H, Hiyama T, Tanaka S, et al: Risk factors for hepatocellular carcinoma among patients with chronic liver disease. N Engl J Med. 1993; 328: 1797-1801. 10.1056/NEJM199306243282501 7684822

